# Circadian Rhythms of PER2::LUC in Individual Primary Mouse Hepatocytes and Cultures

**DOI:** 10.1371/journal.pone.0087573

**Published:** 2014-02-03

**Authors:** Casey J. Guenthner, Martha E. Luitje, Lorna A. Pyle, Penny C. Molyneux, Jimmy K. Yu, Alexander S. Li, Tanya L. Leise, Mary E. Harrington

**Affiliations:** 1 Neuroscience Program, Smith College, Northampton, Massachusetts, United States of America; 2 Department of Mathematics, Amherst College, Amherst, Massachusetts, United States of America; University of Texas Southwestern Medical Center, United States of America

## Abstract

**Background:**

Hepatocytes, the parenchymal cells of the liver, express core clock genes, such as *Period2* and *Cryptochrome2*, which are involved in the transcriptional/translational feedback loop of the circadian clock. Whether or not the liver is capable of sustaining rhythms independent of a central pacemaker is controversial. Whether and how circadian information may be shared among cells in the liver in order to sustain oscillations is currently unknown.

**Results:**

In this study we isolated primary hepatocytes from transgenic *Per2^Luc^* mice and used bioluminescence as a read-out of the state of the circadian clock. Hepatocytes cultured in a collagen gel sandwich configuration exhibited persistent circadian rhythms for several weeks. The amplitude of the rhythms damped, but medium changes consistently reset the phase and amplitude of the cultures. *Cry2^−/−^ Per2^Luc^* cells oscillated robustly and expressed a longer period. Co-culturing with wildtype cells did not significantly shorten the period, indicating that coupling among hepatocytes is insufficient to synchronize cells with significantly differing periods. However, spatial patterns revealed by cellular imaging of wildtype cultures provided evidence of weak local coupling among the hepatocytes.

**Conclusions:**

Our results with primary hepatocyte cultures demonstrate that cultured hepatocytes are weakly coupled. While this coupling is not sufficient to sustain global synchrony, it does increase local synchrony, which may stabilize the circadian rhythms of peripheral oscillators, such as the liver, against noise in the entraining signals.

## Introduction

Circadian or daily rhythms are internally regulated by the hypothalamic suprachiasmatic nucleus (SCN) and are externally entrained by environmental factors such as light and food intake [1]. Cells throughout the body can generate circadian oscillations using transcriptional-translational feedback loops involving several genes, including *Period2* (*Per2*) and *Cryptochrome 2* (*Cry2*) on the negative limb and *Bmal1* on the positive limb of the main feedback loop [1]. It is thought that the SCN orchestrates circadian rhythms throughout the body, setting the phases of a widely distributed network of cellular oscillators by regulating the autonomic nervous system [2] and by outputs via neural and humoral paths [1]. Maintenance of internal temporal order is critical for positive health outcomes and successful aging [3].

Prior research suggests that the liver may be able to maintain circadian rhythms independently of the SCN, but this research has not been conclusive. Feeding mice or rats during their normal fasting time can entrain the circadian rhythms of the liver without shifting the SCN clock [4], [5], suggesting that *in vivo* the liver may be able to hold a phase independent from the SCN. Yoo et al. [6] showed that liver explants from *Per2^Luc^* transgenic mice remained rhythmic for more than 20 days *in vitro*, undisturbed, and rhythmicity was observed even when liver explants were prepared from SCN-ablated animals. However, Guo et al. [7] argue that culture preparation may have synchronized these liver explants (a premise supported by [8]). In an experiment measuring the ratio of *Per:Bmal1* expression in liver of SCN-ablated hamsters Guo et al. [7] observed less variability than would be expected in this ratio if liver were able to maintain a free-running rhythm *in vivo*. Recent advances allow *in vivo* imaging of gene expression in the liver and demonstrate that the liver can support circadian cycles even in SCN-ablated mice [8], [9]. These studies must be interpreted with caution, since it is possible that surgery may have synchronized the liver oscillators in [9], and in [8] only one circadian cycle was observed, with injections of luciferin and anesthesia at each measurement time point. It is therefore possible, but uncertain at this time, that hepatocytes can act as coupled oscillators, sharing circadian information with other hepatocytes and thus enabling the sustained rhythms observed in the isolated liver.

A novel approach to addressing this question is to use cultured hepatocytes. In this study we isolated primary hepatocytes from transgenic mice expressing a fusion protein of PERIOD2 and LUCIFERASE (PER2::LUC), providing a bioluminescent read-out of the circadian clock. We cultured them in a collagen gel sandwich configuration, which allows a layer of cells to maintain polarity and the differentiated hepatocyte phenotype. Cultures of hepatocytes in the collagen gel sandwich configuration express tight and gap junctions and maintain hepatocyte-specific functions, such as albumin and urea secretion, for several weeks [10]. Hepatocytes cultured on a single layer of collagen without an overlying gel show rhythms lasting only a few days [11]–[13]. Prior research suggests that culture conditions affect coupling and the robustness of expressed rhythms in fibroblasts [14]. Here we demonstrate sustained circadian rhythms of hepatocytes cultured in the collagen gel sandwich configuration.

To test if hepatocytes communicate circadian phase, we co-cultured hepatocytes from wildtype (WT) mice with those from longer period *Cry2^−/−^* mice, to produce mixed cultures in which only the *Cry2^−/−^* hepatocytes were bioluminescent. In further experiments, we imaged hepatocyte cultures to examine circadian oscillations of individual cells, and we determined that the cellular rhythms remain closer in phase than would be expected for uncoupled cells. Simulations tailored to the observed locations, phases and periods of cells in each imaged culture provide additional evidence of weak local coupling. Such weak local coupling may help stabilize the circadian rhythm of the liver but is insufficient to globally synchronize the hepatocyte cultures.

## Results

### Primary hepatocytes display persistent circadian rhythms in culture

**Figure 1:**
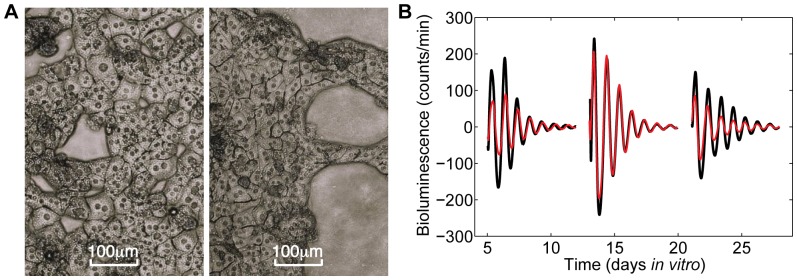
Representative cultures. **A**) Phase contrast micrographs of primary hepatocytes in two representative collagen gel sandwich cultures on DIV 1 (on left) and DIV 9 (on right), with scale bars representing 100 µm. B) Baseline-subtracted PER2::LUC bioluminescence from two representative hepatocyte cultures plated at 1×10^6^/mL with medium changes timed to allow periodic 24 h urea assays.

Use of an established two-step collagenase isolation technique [15] yields cultures of hepatocytes that can be distinguished by a characteristic cuboidal morphology and attachment to other cells that develops over time *in vitro* (see Figure 1A and Movie S1). The use of a collagen gel sandwich configuration further preserves morphology and function of isolated hepatocytes [10].

In order to evaluate the long-term function of our cultures, we monitored urea excretion, an indicator of hepatocyte function, over 4 weeks following culture preparation. In an initial set of experiments, collagen gel sandwich hepatocyte cultures were observed over 3 weeks from day *in vitro* 5 (DIV 5) to DIV 28 with medium changes on DIV 4, 5, 12, 13, 20, 21 and 28, 29. Medium was collected and assayed for 24 h urea content (using kit from Stanbio Urea Nitrogen (BUN), Stanbio Laboratory, Boerne, TX). Urea formation in the liver is important in the metabolism of amino acids and the proteins involved in urea formation have been shown to display circadian rhythmicity [16]. Mean urea output was 97, 70, 34, and 43 µg/million cells/day on DIV 4–5, 12–13, 20–21, and 28–29, respectively (n = 45 cultures). Thus, urea formation declines shortly after culturing but stabilizes after approximately three weeks in culture, consistent with observations in rat hepatocytes cultured under similar conditions [17].

Circadian rhythms in PER2::LUC bioluminescence of the cultures remained consistent between weekly medium changes over three weeks (Figure 1B). The periods were not statistically different between weeks (one-way repeated measures ANOVA, F(2,24) = 0.20, p = 0.82). The mean period was 23.6 h with a standard deviation of 0.5 h (13 cultures derived from 2 animals, measured over 3 weeks). The ratio of autocorrelation half-life to period, which measures stability of amplitude and synchronization within each culture, improved from 0.92±1.7 cycles during week 1 to 1.04±0.13 cycles during week 2 to 1.12±0.19 cycles during week 3 (mean±standard deviation; significant difference according to one-way repeated measures ANOVA, F(2,24) = 5.4, p = 0.01), indicating that the cultures' rhythms remained stable or even improved over the course of the 3 weeks.

### Medium changes reset the phase of cultures

**Figure 2:**
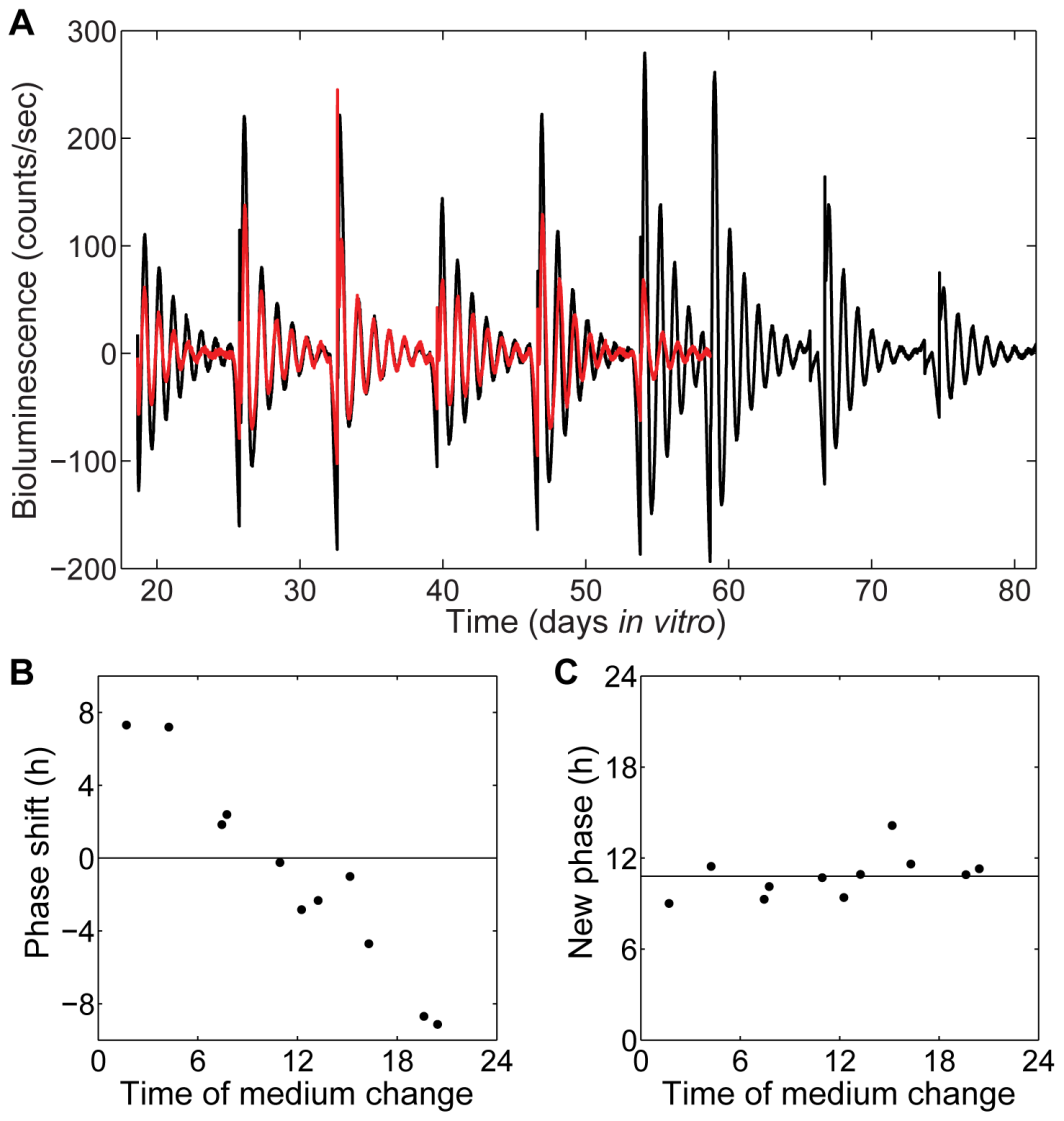
Medium changes reset the phase of PER2::LUC rhythms. **A**) Baseline-subtracted PER2::LUC bioluminescence traces for two long-term collagen gel sandwich cultures maintained with weekly medium changes. All phase shift data were generated from these two traces. **B**) Phase response curve showing phase shift in the PER2::LUC rhythm in response to a medium change given at the indicated times in hours after predicted acrophase. A positive phase shift indicates an advance of the phase, while a negative phase shift indicates a delay. **C**) Phase transition curve showing the new phase of the PER2::LUC rhythm (at the time of medium change, where phase 0 indicates acrophase) following a medium change given at the indicated times in hours after predicted acrophase.

Previous work has shown that peripheral oscillators such as fibroblasts are sensitive to medium changes, which can synchronize the circadian rhythms of cells in culture [18]. To determine whether medium changes also have a synchronizing effect on hepatocyte cultures, two cultures were maintained and bioluminescence measured over DIV 18–58 or 18–81, shown in Figure 2A. Medium changes occurring 1 to 12 h after the peak of PER2::LUC expression advanced the phase of the rhythms, whereas medium changes at other times delayed the rhythm (Figure 2B). The medium change reset the phase to be roughly 11 h after acrophase, regardless of the phase the culture was in at the time of the medium change (Figure 2C). We find that medium changes do provide a strong resetting signal that synchronizes the phases of the hepatocytes and restores the amplitude.

### *Cry2^−/−^* hepatocytes exhibit longer period oscillations than wildtype hepatocytes, with reduced damping

**Figure 3:**
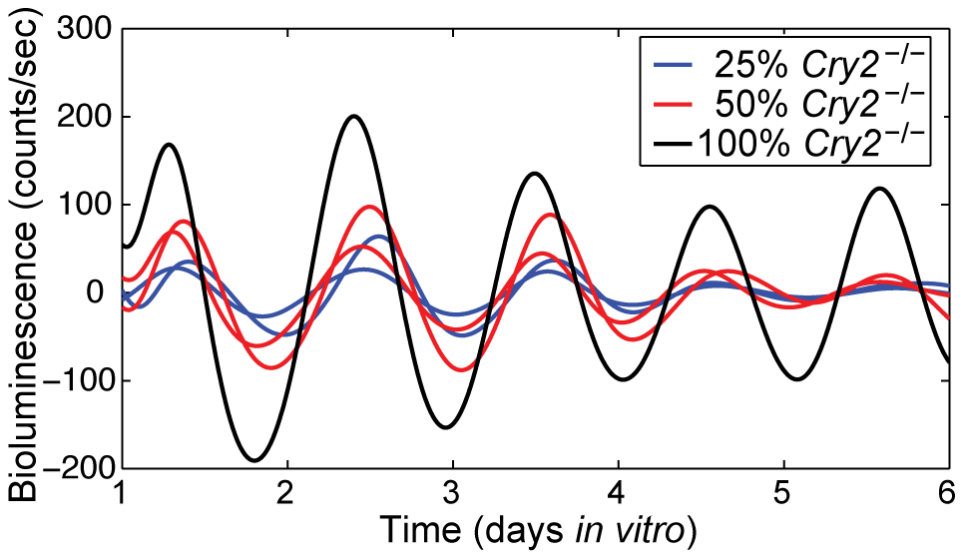
PER2::LUC bioluminescence traces of *Cry2^−/−^ Per2^Luc^* hepatocytes co-cultured with WT hepatocytes. Representative traces from Isolation D with proportions of 100%, 50%, and 25% *Cry2^−/−^ Per2^Luc^* cells (see Table 1).

In order to examine interactions between hepatocytes with different circadian characteristics, we first characterized circadian rhythms in hepatocytes isolated from mice with a key circadian clock gene, *Cry2*, knocked out. Liu et al. [19] reported that individual *Cry2^−/−^* SCN neurons and fibroblasts oscillate with a longer period than wildtype (WT) cells and that a higher proportion of *Cry2^−/−^* cells are rhythmic than are WT in both cell types. In addition, they found that *Cry2^−/−^* fibroblasts exhibited higher amplitude rhythms than WT cells. We isolated primary hepatocytes from *Cry2^−/−^ Per2^Luc^* mice to obtain cultures with a significantly different period; our *Cry2^−/−^ Per2^Luc^* hepatocyte cultures had a period of 29.2±2.3 h (mean±standard deviation; 26 cultures derived from 7 animals, measured over 7 days). The ratio of autocorrelation half-life to period, which measures stability of amplitude and synchronization within each culture, was 1.30±0.43 cycles (mean±standard deviation), significantly greater than the values for the WT *Per2^Luc^* cultures (t-test, p = 0.005 for week 1; p<0.001 if combine *Per2^Luc^* across 3 weeks). Hence the *Cry2^−/−^ Per2^Luc^* cultures damped at a slower rate than the *Per2^Luc^* cultures, consistent with the previous findings from neuronal and fibroblast cultures that *Cry2^−/−^* cells may be more robust oscillators than WT cells. See Figure 3 for representative bioluminescence traces from *Cry2^−/−^ Per2^Luc^* cultures.

### Mixed culture experiments indicate a lack of strong coupling in cultures

To test whether hepatocytes communicate circadian phase, we co-cultured WT cells with either 25% or 50% *Cry2^−/−^ Per2^Luc^* cells. The WT cells were not bioluminescent, so only rhythms in *Cry2^−/−^ Per2^Luc^* cells were recorded (Figure 3). We hypothesized that if cells can synchronize, the mixed cultures should exhibit intermediate periods between those of WT and *Cry2^−/−^*, as has been shown to occur in the SCN and in behavioral rhythms in experiments using chimeric mice in which different proportions of the SCN neurons expressed an altered period due to a mutant *Clock* gene [20]. A similar approach was used by Nagoshi et al. [21] to demonstrate that fibroblasts do not communicate circadian phase to other fibroblasts. In our experiments, variability in period between isolations was high, likely due to differences in the intrinsic circadian period of the individual *Cry2^−/−^* mice used for each isolation; consequently it was not possible to combine data across isolations as originally planned. The periods of WT and *Cry2^−/−^ Per2^Luc^* hepatocytes differ by several hours, so cells must be strongly coupled to overcome this large difference in period and allow a significant period difference to emerge (see modeling discussion below). The within-isolation analysis summarized in Table 1 indicates that strong coupling is not occurring among hepatocytes. However, the mixed culture experiments do not rule out the possibility of weak local coupling among hepatocytes.

**Table 1:** Periods of *Cry2^−/−^ Per2^Luc^* cultures co-cultured with non-bioluminescent WT cells.

Isolation	100% *Cry2* ^−/−^ *Per2^Luc^*	50% *Cry2* ^−/−^ *Per2^Luc^*	25% *Cry2* ^−/−^ *Per2^Luc^*	ANOVA
A	29.7±1.9 h (n = 3)	30.1±1.6 h (n = 5)	27.0±0.5 h (n = 2)	-
B	29.4±1.4 h (n = 2)	30.0±2.2 h (n = 3)	30.7±2.4 h (n = 3)	-
C	30.5±0.9 h (n = 2)	28.7±1.1 h (n = 4)	27.5±1.0 h (n = 4)	-
D	25.5±1.2 h (n = 4)	26.1±0.4 h (n = 12)	26.1±1.4 h (n = 15)	p = 0.57 (F = 0.6)
E	27.3±1.0 h (n = 8)	-	26.4±1.0 h (n = 7)	p = 0.11 (F = 2.9)
F	26.2±0.5 h (n = 4)	-	26.2±0.3 h (n = 4)	p = 0.91 (F = 0.01)

### Cellular imaging of hepatocyte cultures reveals weak local coupling

**Figure 4:**
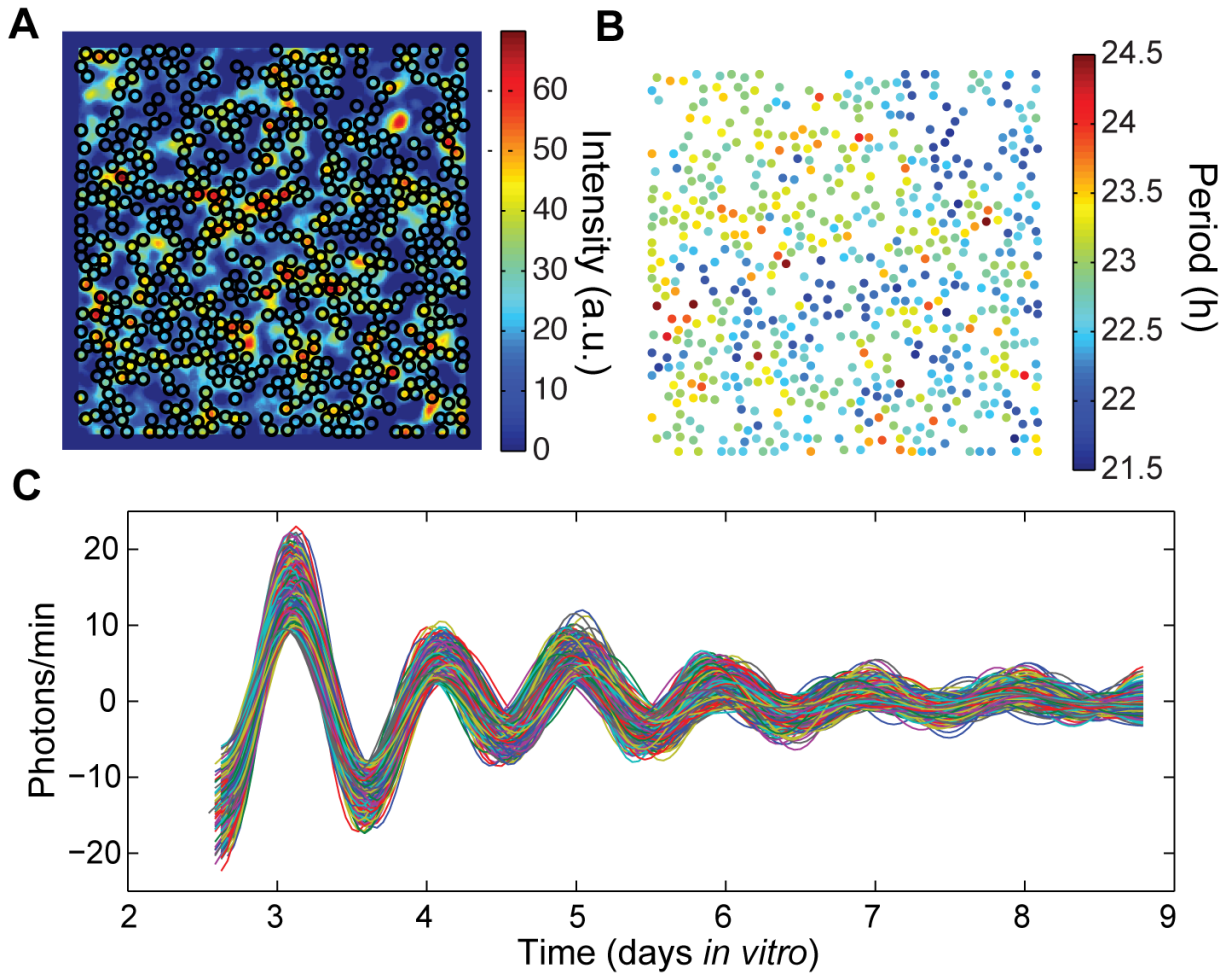
Single cell rhythms in WT *Per2^Luc^* culture. **A**) The intensity of bioluminescence (in arbitrary units) of the field of view captured by the CCD camera over days *in vitro*, as described in the Methods, which was used to determine locations of 630 ROIs in a hepatocyte collagen gel sandwich culture. The average local density is 19 ROIs/mm^2^. **B**) Periods of the rhythmic ROIs (AWT estimate during DIV 3 to 5), with mean 22.8 h and standard deviation 0.5 h. **C**) PER2::LUC bioluminescence traces for the rhythmic ROIs, with trend and noise removed by discrete wavelet transform.

**Figure 5:**
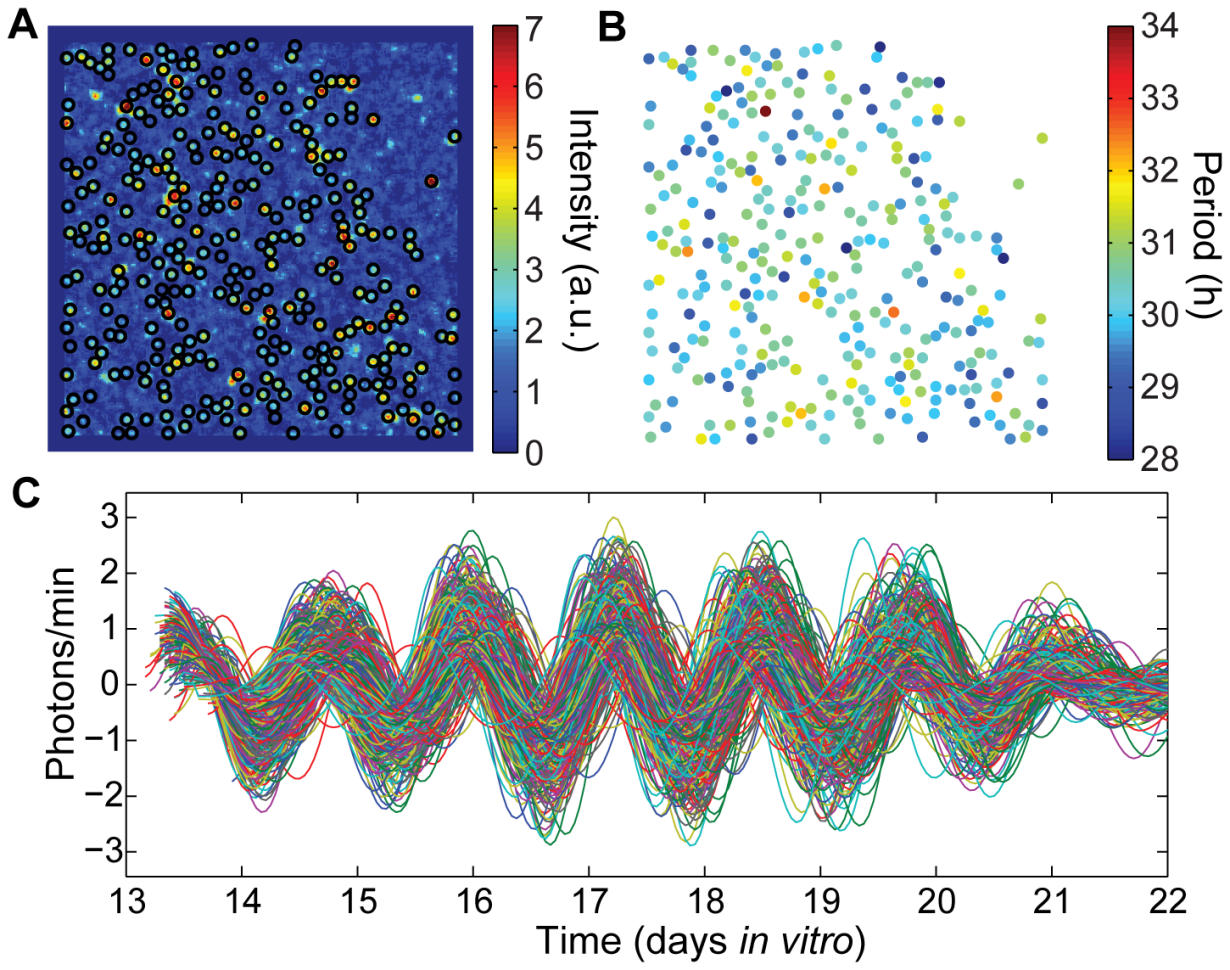
Single cell rhythms in mixed cultures. **A**) The intensity of bioluminescence of the field of view captured by the CCD camera over days *in vitro*, as described in the Methods, which was used to determine locations of 313 ROIs in a hepatocyte collagen gel sandwich culture consisting of 25% *Cry2^−/−^ Per2^Luc^* hepatocytes and 75% WT (non-bioluminescent) cells. **B**) Periods of the rhythmic ROIs (mean AWT estimate during DIV 14–16) with mean 30.2 h and standard deviation 0.8 h. **C**) PER2::LUC bioluminescence traces for the rhythmic ROIs, with trend and noise removed by discrete wavelet transform.

To examine localized effects within the cultures, we imaged bioluminescence from hepatocyte cultures using a high-sensitivity cooled CCD camera. We imaged circadian rhythms from three cultures with 100% WT *Per2^Luc^* cells and from one culture with 25% *Cry^−/−^ Per2^Luc^* cells, as shown in Figures 4 and 5 and Figure S1. Our imaging analysis focused on cell-like regions of interest (ROIs) which likely correspond to individual hepatocytes, identified using a procedure similar to that validated for SCN imaging in [22]; see Methods for further explanation.

Within one of the three 100% WT *Per2^Luc^* cultures reported here, 630 ROIs were identified as rhythmic (Figure 4 and Movie S2, see Figure S1 for the other two cultures). The periods and phases of each ROI at each time point were calculated using the analytic wavelet transform (AWT) as described in the Methods. Because of a medium change before start of recording, the initial phases of the ROIs appear synchronized. More interestingly, the periods of ROIs appear spatially clustered, suggesting local coupling of oscillations (Figure 4). To test the hypothesis that cells are locally coupled, we applied multiple methods for detecting coupling, with analysis of both local and global synchrony in phase or in period. Because circadian data involves phases, we applied circular statistics as described in the Methods section. In particular, we used circular standard deviation as a measure of phase spread.

**Figure 6:**
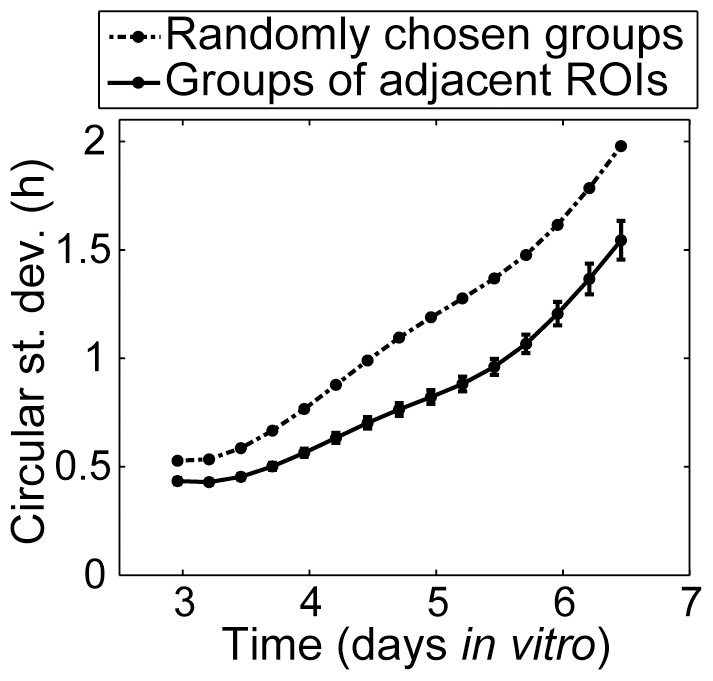
Phase clustering indicating local coupling among single WT *Per2^Luc^* hepatocytes. Circular standard deviation is significantly less in groups of 7 adjacent ROIs than in groups of 7 randomly chosen ROIs, indicating greater synchronization among adjacent ROIs than would occur by chance (p<0.001 at every time point, one-sample z-test using 1,000,000 randomly selected groups to approximate population distribution). Error bars show mean±standard error for the 106 groups of 7 ROIs whose centers lie within a 325-µm diameter circle (ROIs are 78 µm or larger in diameter).

One possible indication of local coupling is spatial clustering of period; coupled cells should exhibit more similar periods than uncoupled cells, with less apparent influence on period as the distance between cells increases. We expect this to be a regional effect, as beyond some threshold distance there is unlikely to be any communication between cells. We did find a modest positive correlation between distance and period difference over all pairs of ROIs within 325 µm of each other in the culture shown in Figure 4A (after subtracting a linear fit with respect to x and y coordinates from the period values to remove the effect of any spatial gradient in the period): r = 0.08, p<0.001 at DIV 3; r = 0.09, p<0.001 at DIV 4; r = 0.13, p<0.001 at DIV 5; and r = 0.10, p<0.001 at DIV 6. We also compared the circular standard deviation of the phases of groups of 7 randomly chosen ROIs to that of groups of 7 adjacent ROIs. Groups of 7 provide a sufficiently large group for stable calculations of the circular standard deviation, yet are small enough to reflect local phenomena. If local coupling is occurring, groups of adjacent ROIs should be significantly closer in phase than randomly chosen groups of ROIs, which is indeed what we observed at all time points, as shown in Figure 6. See Figure S2 for a similar analysis of other WT *Per2^Luc^* cultures.

As an additional test, we examined whether there is a correlation between the local synchronization index *R* (see Methods) in the neighborhood of a cell and how many cells are directly adjacent to that cell. This index provides a measure of how tightly synchronized a set of rhythms is; *R* = 1 for perfectly synchronized phases (all identical) and *R* = 0 for uniformly distributed phases. If local coupling is indeed present, we would expect dense groups of adjacent cells to be more synchronized in phase with each other than sparse groups of spatially separated cells would be. Again we find a modest but significant effect: r = 0.14, p<0.001 at DIV 3; r = 0.13, p<0.001 at DIV 4; r = 0.12, p = 0.002 at DIV 5; r = 0.04, p = 0.3 at DIV 6. After DIV 6, damping of amplitude leads to greater uncertainty in phase determination and consequently a growing instability in the estimated phases.

We can also compare the density of cells in the neighborhood of each cell with the circular standard deviation in ROI phases. To avoid changes due to different numbers of cells, rather than fix the size of the region we fix the number of cells considered to be a “neighborhood” at 5 cells and determine the size of the region required to contain each group of 5 ROIs. We find a negative correlation between density and phase dispersion, as would be expected if local coupling were present (more tightly packed cells should be able to couple more effectively, leading to a narrower set of phases): r = −0.20, p<0.001 at DIV 3; r = −0.17, p<0.001 at DIV 4; r = −0.16, p<0.001 at DIV 5; r = −0.10, p = 0.01 at DIV 6. This effect is reduced if larger sets of cells are considered, indicating that the coupling is highly localized.

In contrast, the 25% *Cry2^−/−^ Per2^Luc^* mixed culture shown in Figure 5 does not exhibit similar evidence of local coupling. In this culture, the long-period *Cry2^−/−^ Per2^Luc^* cells are distributed among WT cells (that are not bioluminescent), and any weak local coupling that may be present is insufficient to synchronize across the 3–4 h period difference, consistent with the results from our whole-field mixed culture recordings.

### Modeling supports the hypothesis of weak local coupling among cultured hepatocytes

**Figure 7:**
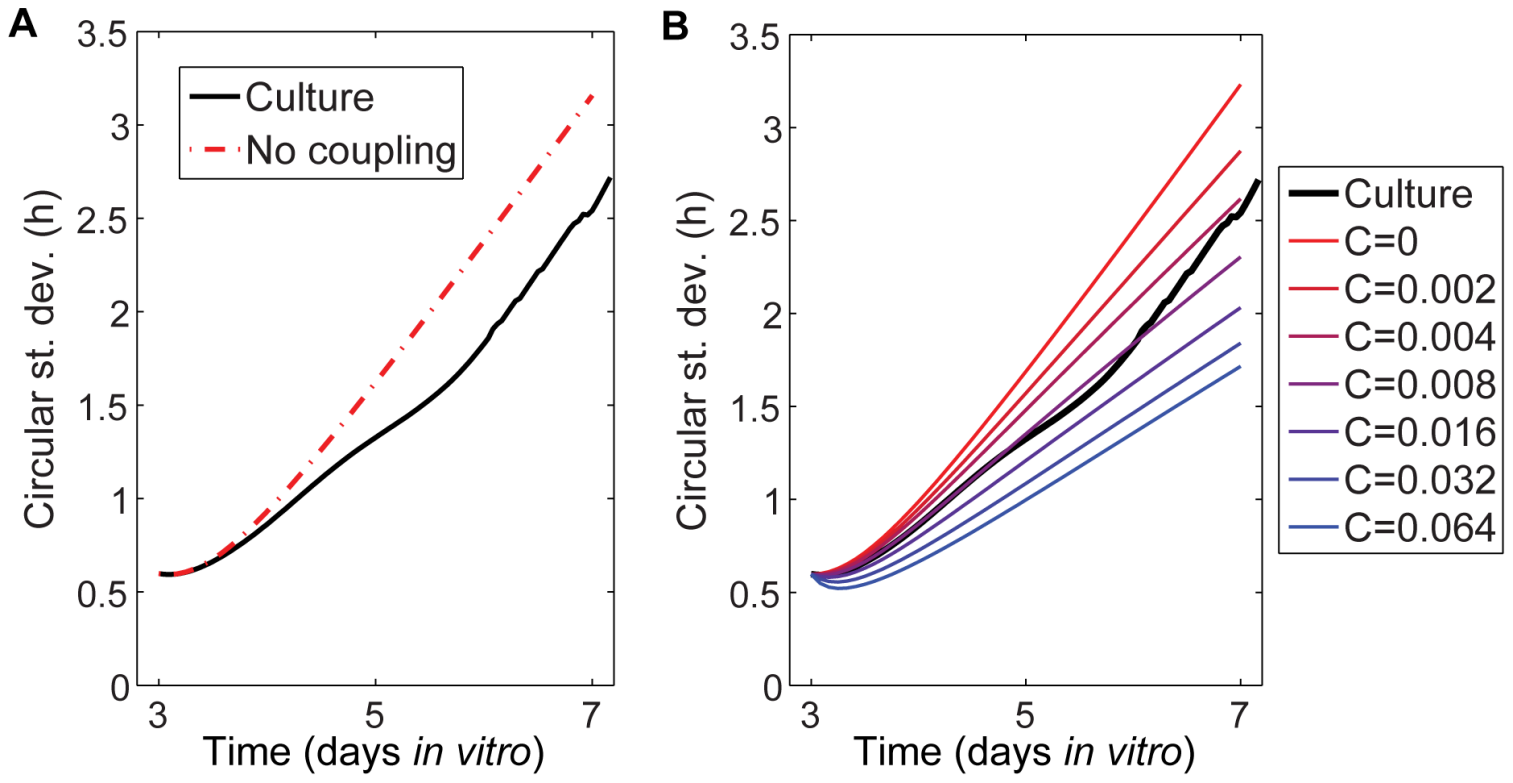
Comparison of WT *Per2^Luc^* culture and simulations. **A**) Comparison of phase spread over time observed in culture ROIs with phase spread expected if no coupling were present (corresponding to *C* = 0 in the Kuramoto model). Initial phases and periods of the cells in the model are set equal to those on DIV 3 for the culture, so the phases in the model and culture agree initially but diverge after around 12 h. **B**). Comparison of culture with simulations of locally coupled oscillators with randomly permuted periods and indicated coupling strengths (average over 25 simulations for each coupling strength). After DIV 6, damping of the amplitude leads to greater uncertainty in phase determination and consequently increased phase spread.

To further test the hypothesis of local coupling and estimate its strength, we can compare the global synchronization of phases over time in the culture to that predicted by a phase-only model of locally coupled oscillators, using experimentally determined values of initial phases, periods, and cell locations (see Methods for details). We compared the experimentally observed phase distributions over time for the WT *Per2^Luc^* culture shown in Figure 4 to those predicted by the model. The dashed curve in Figure 7A corresponds to no coupling (*C* = 0) so that phases are steadily drifting apart according to their intrinsic periods (see Figure S3 for a similar analysis of the other two cultures). The culture's standard deviation of phase increases more slowly than the prediction for no coupling, suggesting that some factor is present to slow the spreading of ROI phases. This implies that the observed period over time for each ROI is somewhat different from its intrinsic period, as it will include the coupling effects. Directly inferring model parameters, such as coupling strength and intrinsic frequencies of the cells from the imaging data is difficult because overall period in the culture tends to change over time at a variable rate, and relatively few cycles are available. However, because the coupling is weak and localized, by permuting the observed ROI periods, we can undo the subtle localized effects of coupling on the ROI periods and hence simulate the hepatocyte culture using experimentally determined period values and cell locations. The only remaining parameter to determine is the coupling strength *C*, which we can indirectly infer by comparing simulations to the experimental values. Based on the modeling results shown in Figure 7B, the coupling strength *C* is roughly 0.008 hr^−1^, weak but positive. In comparison, for local clusters of 5 ROIs to synchronize their periods, a coupling strength of at least *C* = 0.017 hr^−1^ is required; because of the culture's highly sparse connectivity, global synchrony is only attainable with an extremely strong coupling strength several orders of magnitude greater than that needed for regional synchronization. The prediction of local-only coupling that is weak in strength is consistent with the modest correlations observed in the local coupling analysis given above.

**Figure 8:**
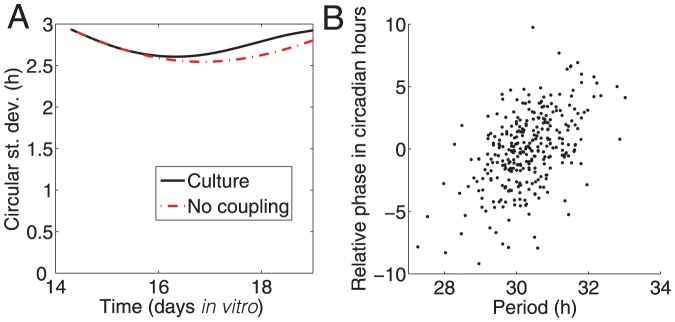
Phase and period analysis of *Cry2^−/−^ Per2^Luc^* mixed culture. **A**) Comparison of the circular standard deviation of ROI phases over time observed in the mixed culture shown in Figure 5 with the circular standard deviation in phases expected if no coupling were present. Initial phases and periods of the cells in the model are set equal to those on DIV 14 for the culture. **B**) Periods and phases at DIV 14 are positively correlated in this mixed culture (r = 0.49, p<0.001), explaining why the circular standard deviation initially decreases despite the lack of coupling, before gradually increasing again. ROIs with shorter periods are initially later in phase, but over time advance in phase compared to ROIs with longer periods, causing the ROIs to appear to come together in phase over the DIV 14–16, but then spread apart afterward.

The weak local coupling present in the hepatocyte cultures is insufficient to overcome the period difference of several hours occurring in the 25% *Cry2^−/−^ Per2^Luc^* mixed cultures. The synchronization over time of ROIs in the mixed culture is consistent with what would be expected if no local coupling were occurring. The strength of the local coupling is small compared with the period difference between the cell types, so its effect on phase is not detectable. The circular standard deviation shown in Figure 8A initially decreases, suggesting a possible synchronizing effect among hepatocytes in the mixed culture. However, examination of the initial phases, shown in Figure 8B, reveals that the initial improvement in synchronization is a consequence of shorter period ROIs starting with phases that lag behind the longer period ROIs. For the first 2 days *in vitro*, the phases drift closer together, before passing each other and drifting apart again.

Modeling with locally coupled oscillators suggests that for a significant difference in period to emerge in the mixed culture experiments, the coupling would have to be much greater than that observed. For instance, simulations like those shown in Figure S4 indicate that to detect a significant shortening of the period in a mixed culture, the coupling strength would have to be roughly 4 times greater than what is observed in our experiments.

## Discussion

Prior research suggests that the liver can oscillate independently of the main circadian pacemaker, the suprachiasmatic nucleus in the hypothalamus of the brain, in certain circumstances. Yet the question remains, do the cells of the liver bring their individual rhythms into synchrony with each other and act as coupled oscillators?

This broadens into a larger question in circadian biology: do peripheral oscillators ever show the capability to act as coupled oscillators? Prior studies with fibroblast cultures strongly suggest that fibroblasts are independent circadian oscillators that do not communicate circadian information [22], [24], although a constitutive diffusible signal appears necessary to sustain rhythmicity in fibroblasts [23]. On the other hand, the ability of SCN neurons to couple to each other is crucial for the function of the central pacemaker [24]. We present results here suggesting that liver cells, unlike fibroblasts, may communicate circadian information such as phase to nearby cells, but in a much weaker and more localized manner than occurs among neurons in the SCN. Hepatocytes cultured in a collagen gel sandwich configuration cycled with persistent circadian rhythms for several weeks with medium changes. The amplitude of the rhythm damped over the week, but medium changes reset the phase and amplitude. Similar phase synchronization via resetting by a single pulse has also been observed in cultured fibroblasts using a dexamethasone pulse [21] and in TTX-treated SCN neurons (to decouple) using a temperature pulse [25].

To investigate whether hepatocytes may act as coupled oscillators, we cultured cells from mutant mice with a longer period circadian rhythm and determined that *Cry2^−/−^ Per2^Luc^* hepatocytes oscillated robustly and expressed a longer period. Interestingly, co-culturing with wildtype cells did not significantly shorten the period of the *Cry2^−/−^ Per2^Luc^* hepatocytes, indicating that the coupling among hepatocytes was insufficient to synchronize cells with significantly differing periods. However, spatial patterns revealed by cellular imaging of wildtype cultures provided evidence of weak local coupling among the hepatocytes. We conclude that cultured hepatocytes are weakly coupled circadian oscillators. While this coupling is not sufficient to sustain global synchrony, it does increase local synchrony, which may stabilize the circadian rhythms of peripheral oscillators like the liver against noise in the entraining signals. Brain-specific rescue of circadian rhythmicity in ClockΔ19 mice also restored rhythmicity to the liver but with reduced amplitude [26], so local coupling may also act to enhance the amplitude of circadian oscillations in peripheral tissues.

The rhythms we observed from our cultures damped over time *in vitro*. This damping could potentially be explained by loss of amplitude in individual hepatocytes and/or loss of synchrony among the population of hepatocytes. Of course, these may not necessarily reflect intrinsic properties of hepatocytes but may depend on our experimental conditions. It is possible that these results would differ under different culture conditions [14], [27]. Our imaging experiment measuring rhythms of individual hepatocytes suggested that the damping we observed across entire cultures was a reflection of damping of rhythms of individual hepatocytes, so the damping of the rhythm of the entire culture may not be primarily due to a loss of synchrony. On the other hand, cultured fibroblasts can show robust individual cellular rhythms but quickly fall out of phase following a synchronizing medium change or dexamethasone pulse so that the culture's overall rhythm appears damped [18], [21].

When assessing whether coupling might be present in the hepatocyte cultures, we applied multiple methods to minimize the chance of a false positive. Subtle external synchronizing influences on the culture could account for the global synchrony being greater than would be expected if there were no coupling (Figure 7A), but would be unlikely to cause the localized increase in synchrony observed in Figure 6. On the other hand, the apparent local coupling effects we observe could possibly be an artifact of the ROI selection, but that would not explain the greater than expected global synchrony in Figure 7A.

The mathematical modeling provides further evidence supporting the statistical analysis, demonstrating the presence of weak local coupling that influences cell phases but is insufficient to globally synchronize the culture. Similarly, Rougemont and Naef [28] used a phase-only coupled oscillators model incorporating stochastic fluctuations in the intrinsic frequency to analyze the fibroblasts in [21] and [18]. They found that the coupling strength present in the fibroblasts was half of that required to induce synchrony in the culture, under the assumption of all-to-all coupling. Under our local coupling assumption, we found that the hepatocyte coupling strength is roughly half that required for local synchrony.

The estimated strength of the local coupling that we observed for hepatocytes in culture is likely weaker than that for liver tissue. Gaps that occur in two-dimensional culture can result in a sparse network formed by adjacent cells, with each cell likely only coupled to a few other cells. Liver tissue may support much stronger coupling due to its three-dimensional structure and the possibility of physiological coupling mechanisms not present in the hepatocyte culture. Additionally, the liver *in vivo* is exposed to systemic cues such as body temperature fluctuations and serum factors that can serve as entraining cues [29], [30]. Thus the weak local coupling observed in the *in vitro* hepatocyte culture represents a lower bound for what may occur *in vivo* in liver tissue.

The coupling signal(s) in the hepatocyte culture could be either a cytoplasmic factor that diffuses to neighboring cells through gap junctions or a locally diffusing excreted factor. Hepatocyte gap junctions are potentially permeable to small signaling molecules, including Ca^2+^ and cAMP, that are known to regulate circadian rhythms in other cell types [31], [32]). Regulation of circadian rhythmicity by excreted factors has also been demonstrated in other cell types. For instance, in fibroblasts, paracrine signaling is essential for maintenance of rhythmicity, but these signals do not have to be rhythmic and do not appear to influence circadian period; in particular, co-culturing PER2::LUC wildtype fibroblasts with non-luminescent *Bmal1* ^−/−^ or *Cry2* ^−/−^ fibroblasts to achieve a high-density culture enhanced the rhythmicity of the WT cells but did not affect their period [23]. In contrast, potential paracrine regulators of local coupling in hepatocyte cultures must be circadianly regulated and capable of influencing circadian phase or period. As many as 20% of soluble proteins in mouse liver are regulated in a circadian manner [16]; circadian rhythms in the abundance of diffusible excreted proteins or enzymes that synthesize diffusible small molecules suggest numerous potential pathways through which cell-cell circadian coupling could be orchestrated. Testing these possibilities and characterizing the mechanisms of local coupling will be an important goal for future work.

These results expand our understanding of the regulation of circadian rhythms in the liver, rhythms that are important in liver nutrient metabolism, drug detoxification, and many other functions [33]. More than 10% of the liver transcriptome and proteome are regulated in a circadian manner [16], [34], [35]. Rhythmicity of genes involved in metabolism and detoxification depend on the hepatocyte clock [36] and mice without a functioning hepatocyte circadian clock show hypoglycemia during the fasting phase of the diurnal cycle [37]. Circadian rhythm disruption has a large impact on metabolism, and the liver is a likely target for those negative health effects. Circadian rhythms of the liver can be synchronized by humoral and neural outputs from the SCN [38], as well as by body temperature and feed-fast cycles [4], [5], [36], [39], [40]. Our demonstration that a medium change resets and restores the rhythm of cultured hepatocytes allows any researcher to set the circadian phase of their cultures and then conduct experiments to determine if circadian phase influences dependent measures. Our studies establish that hepatocytes may provide each other local information relevant to circadian phase and period.

## Methods

### Ethics Statement

Animal welfare laws were followed and all protocols were approved by the Smith College Institutional Animal Care and Use Committee.

### Animals

Three types of mice on a C57Bl6/J background were used: wildtype *Per2^Luciferase^* animals, which carry a PER2::LUC fusion protein for bioluminescent reporting (http://jaxmice.jax.org/strain/006852.html), developed by [6]; *Cry2^−/−^ Per2^Luc^* animals in which the*Cry2* gene is knocked out [41], and wildtype C57Bl/6J animals. Breeding colonies were maintained in the Animal Care Facility at Smith College, under a 12∶12 light∶dark cycle. The *Cry2^−/−^ Per2^Luc^* animals were derived from mice developed by Dr. G.T.J. van der Horst (Erasmus MC, The Netherlands) and were acquired from Dr. D.K. Welsh (UCSD, San Diego, CA). C57Bl6/J mice were from an in-house breeding colony as were WT *Per2^Luc^* mice, founders provided by Dr. J. Takahashi, from mice backcrossed 7–11 generations. Male or female animals of 1–4 months of age were used, and for cultures of mixed genotypes, animals were matched by sex and age.

### Hepatocyte isolation and culture

Hepatocytes were isolated using a 2-step perfusion method [15], further modified to improve sustained viability in murine hepatocytes (detailed protocol available upon request.) Animals received an intraperitoneal injection of a ketamine/xylazine solution (ketamine, 200 mg/kg; xylazine, 20 mg/kg, in 0.2 mL saline.) The liver was perfused *in situ* through the hepatic portal vein with 50 mL of an EGTA solution (in g/L: 0.1902 EGTA, 8.0 NaCl, 0.4 KCl, 6.0 HEPES, 0.06 KH_2_PO_4_, 0.35 NaHCO_3_, 1.0 d-glucose, pH 7.4) followed by 80–90 mLs of collagenase solution containing low-glucose DMEM (Gibco) supplemented with 600 mM HEPES and antibiotics (penicillin/streptomycin, 2.5 mL/L, Gibco), and containing equal parts of Type III and Type IV collagenase (Worthington Biochemical) @100 U/mL. All solutions were maintained at 37°C throughout the isolation and the collagenase solution was heated at 37°C for 90 minutes before use. After digestion and separation from the liver capsule by gentle shaking in additional collagenase solution, the cell solution was passed through a 100 um filter and centrifuged for 2 min at 50 g and then additionally 2×2 min @ 50 g in wash buffer (Williams Medium E with 5% fetal bovine serum.) Cells were resuspended in a plating solution of Williams Medium E, 5% fetal bovine serum, dexamethasone and a cocktail solution of penicillin-streptomycin, bovine insulin, GlutaMAX™ and HEPES (Gibco) and placed on ice until plating. Routinely, 30–40 million cells were obtained with viability between 85 and 94%, as determined by trypan blue exclusion.

Collagen gel sandwich cultures were prepared by spreading 400 uL of a freshly prepared collagen solution [1 part 10× DMEM, (Gibco, at pH 7.4) and 9 parts collagen (BD Biosciences, at 1.25 mg/mL)] over 35-mm tissue culture dishes and allowing the gel layer to dry at 37°C for 30 min. 1.25 million hepatocytes were seeded in 1.25 mL of plating medium and allowed to attach for 1½ hours at 37°C and 10% CO_2_. Cultures were aspirated and washed briefly with wash buffer, then 1 mL maintenance medium [Williams Medium E, dexamethasone, and a cocktail solution of penicillin-streptomycin, insulin, transferrin, selenium complex, BSA and linoleic acid, GlutaMAX™ and HEPES (Gibco)] was added to each dish. Dishes were maintained at 37°C and 10% CO_2_, with a medium change after 24 hours. The top layer of the collagen gel sandwich was added after 48 hours at 400 uL per dish. After drying for 1½ hours medium was replaced, with 1 mL fresh maintenance medium for continued culture in a CO_2_ incubator, or the culture was sealed with 2 mL culture medium with 1.5 g/L NaHCO_3_ and 100 mM luciferin (Promega) added for measurements of bioluminescence and imaging. Dishes were sealed with silicone grease (Dow-Corning) and circular glass coverslips (Erie Scientific).

### Measures of bioluminescence

Bioluminescence monitoring of an entire culture was measured using a LumiCycle photomultiplier tube detector system (Actimetrics, Wilmette, IL) at 37°C. Bioluminescence imaging was performed using a Nikon inverted microscope in a dark room with a heater chamber kept at 37°C. Images were collected using a Nikon CFI Plan Apo 4× objective (bioluminescence) and transmitted to a CCD camera (Andor IKon DU934N-BV) cooled to −95°C. Signal-to-noise ratio was improved by using 4×4 binning of pixels for bioluminescent imaging and images of 60 min duration were collected continuously.

### Analysis

The LumiCycle's photomultiplier tubes detected and amplified the photons emitted from each whole culture dish once every ten minutes and generated bioluminescence over time data in counts per second. LumiCycle Analysis software (Actimetrics, Wilmette, IL) was used to analyze the raw bioluminescence data: the baseline was subtracted using a 24 h running average, the noise was smoothed using a 2 h running average, and a damped sine wave was then fit using the Levenberg-Marquardt algorithm (Figures 1 and 2). The phase responses to medium changes (Figure 2) were analyzed from the long-term collagen gel sandwich cultures by plotting the baseline subtracted and smoothed LumiCycle data onto an actogram using ClockLab software (Actimetrics), and using regression lines fit to the acrophases, dropping the day of the medium change, to find the phase shift.

### Imaging

Bioluminescence data were gathered over 7 days in images with one-hour exposures and the image sequence was analyzed to remove noise, identify ROIs and determine circadian parameters. The image sequences are processed by removing cosmic ray noise via thresholding at the 99^th^ percentile, and subtracting background (value in empty areas). In addition, each image is spatially smoothed to reduce noise by convolving locally with a Gaussian. Each pixel in the images corresponds to 13 µm. ROIs are determined iteratively, similar to the procedure described in [22]: An “intensity” matrix (shown in Figure 4A) is generated as the product of summed images from the 1^st^ to 2^nd^ field peak and the amplitude over the 1^st^ cycle (to highlight bright areas that may be rhythmic). At each step, the current brightest spot is found in the intensity matrix, around which a 6-pixel radius disk is then set to 0 to enforce spacing of ROIs, with the process repeating until the intensity matrix is zeroed out. For each bright spot, the time series is read off the processed image sequence and an analytic wavelet transform as described in [42] and [43] was used to determine period and phase at each time point. An ROI is considered rhythmic if its period is between 18 and 36 hours and the SNR (the logarithm of the ratio of the energy in the signal to that of the noise) is greater than 0, so that the oscillation can be reliably detected above the noise. Candidate ROIs not meeting these requirements were rejected and not included in the ROI analysis.

### Modeling

To simulate the circadian oscillations in a hepatocyte culture, we use a Kuramoto model with local coupling:
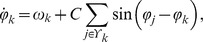
where 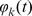 is the phase of cell *k* in radians at time *t*, 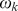 is its intrinsic frequency (period is 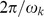), 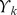 is the set of cells coupled to cell *k*, and *C* is the coupling strength. The instantaneous phases and periods in the culture 24 h after start of recording were used as initial conditions (to avoid edge effects in AWT calculations). Based on examination of cell locations in brightfield images of hepatocyte cultures (e.g. Fig. 1A), we assume ROIs are coupled if their centers are less than 130 µm apart (where ROIs have diameter 78 µm or greater, with centers located as in Figure 4A), with most ROIs coupled to 0–5 other ROIs (median of 3).

### Circular statistics

We used the following circular statistics definitions: The *circadian mean* 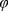 of a set of phases 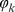 given in radians is 
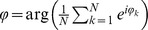
. The *synchronization index* is 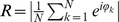, and the *circular standard deviation* is 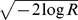. Multiply by 12/π to convert phases from radians to circadian hours.

### Computation

Custom scripts were developed for data analysis in MATLAB R2013a (The Mathworks, Inc). Wavelet analysis used the freely available toolboxes *jlab* 0.94 (J.M. Lilly, http://www.jmlilly.net/jmlsoft.html) and *wmtsa* (C. Cornish, http://www.atmos.washington.edu/~wmtsa/) and was carried out as described in [42], [43].

Simulations of the locally coupled Kuramoto model were run in MATLAB R2013a using ode45 with a minimum time step of 0.2 h.

## Supporting Information

Figure S1 **Examples of other cultures.** **A**) Locations of 288 rhythmic ROIs in a WT *Per2^Luc^* hepatocyte culture with average local density around cells of roughly 12 cells/mm^2^. **C**) Locations of 497 rhythmic ROIs in another WT *Per2^Luc^* culture with average local density around cells of roughly 15 cells/mm^2^. **B,D**) Periods of the ROIs in (A) and (C).
(TIF)

Figure S2 **Additional examples of localized phase clustering.** **A**) Circular standard deviation over time in groups of adjacent ROIs compared to groups of randomly selected ROIs for the culture shown in Figure S1A. The 12 groups of adjacent ROIs include all possible groups of 7 ROIs lying within a 325-µm diameter circle (ROIs are 78 µm or larger in diameter). **B**) Circular standard deviation over time in 54 groups of adjacent ROIs compared to randomly selected groups for the culture shown in Figure S1B. In both figures, asterisks mark time points at which the mean is significantly different (α = 0.05) according to a one-sample z-test for which 1,000,000 randomly selected groups were sampled to approximate the population distribution.
(TIF)

Figure S3 **Additional examples comparing cultures and simulations.** **A**) Comparison of circular standard deviation in observed ROI phases over time with that expected if no coupling were present (corresponding to *C* = 0 in the model) for the culture shown in Figure S1A. **B**) Similar comparison for the culture shown in Figure S1C. In both graphs, the initial phases and periods of the cells in the model are set equal to those on DIV 3 for the culture, so the phases in the model and culture agree initially but diverge after roughly 12 h.
(TIF)

Figure S4 **Periods of ROIs in mixed culture simulated with different coupling strengths.** **A**) Periods of ROIs at end of 2-week simulation of *Cry2^−/−^ Per2^Luc^* mixed culture with no coupling (*C* = 0). Resulting period of summed rhythm of *Cry2^−/−^ Per2^Luc^* ROIs is 30.2 h. **B**) Simulation with *C* = 0.008, resulting in *Cry2^−/−^ Per2^Luc^* summed rhythm period of 29.8 h. **C**) Simulation with *C* = 0.016, resulting in *Cry2^−/−^ Per2^Luc^* summed rhythm period of 29.5 h. **D**) Simulation with *C* = 0.032, resulting in *Cry2^−/−^ Per2^Luc^* summed rhythm period of 26.8 h. In all simulations, the 313 *Cry2^−/−^ Per2^Luc^* ROIs (large circles) are located as shown in Figure 5, with periods and initial phases as measured from the culture. 583 ROIs mimicking WT (small circles), with period 24.0±1.0 h, were added to simulate a mixed culture. Local coupling has the same form as simulations in Figure 7. However, the local density of cells in these simulations is relatively high, as the spatial distribution of ROIs lacks the physical gaps inherent in the experimental cultures.
(TIF)

Movie S1 **Hepatocytes during first 24 hours post-isolation.** Cells flatten and establish contact with adjacent cells.
(MOV)

Movie S2 **Bioluminescent expression of PER2::LUC in hepatocytes.** Images were collected over DIV 5 through DIV 10, in one-hour bins.
(MOV)
