# Supplementary figures and images for: Circadian Rhythms of PER2::LUC in Individual Primary Mouse Hepatocytes and Cultures
Source: PLoS One. 2014 Feb 3;9(2):e87573. doi: 10.1371/journal.pone.0087573 (PMC3911982; doi:10.1371/journal.pone.0087573)

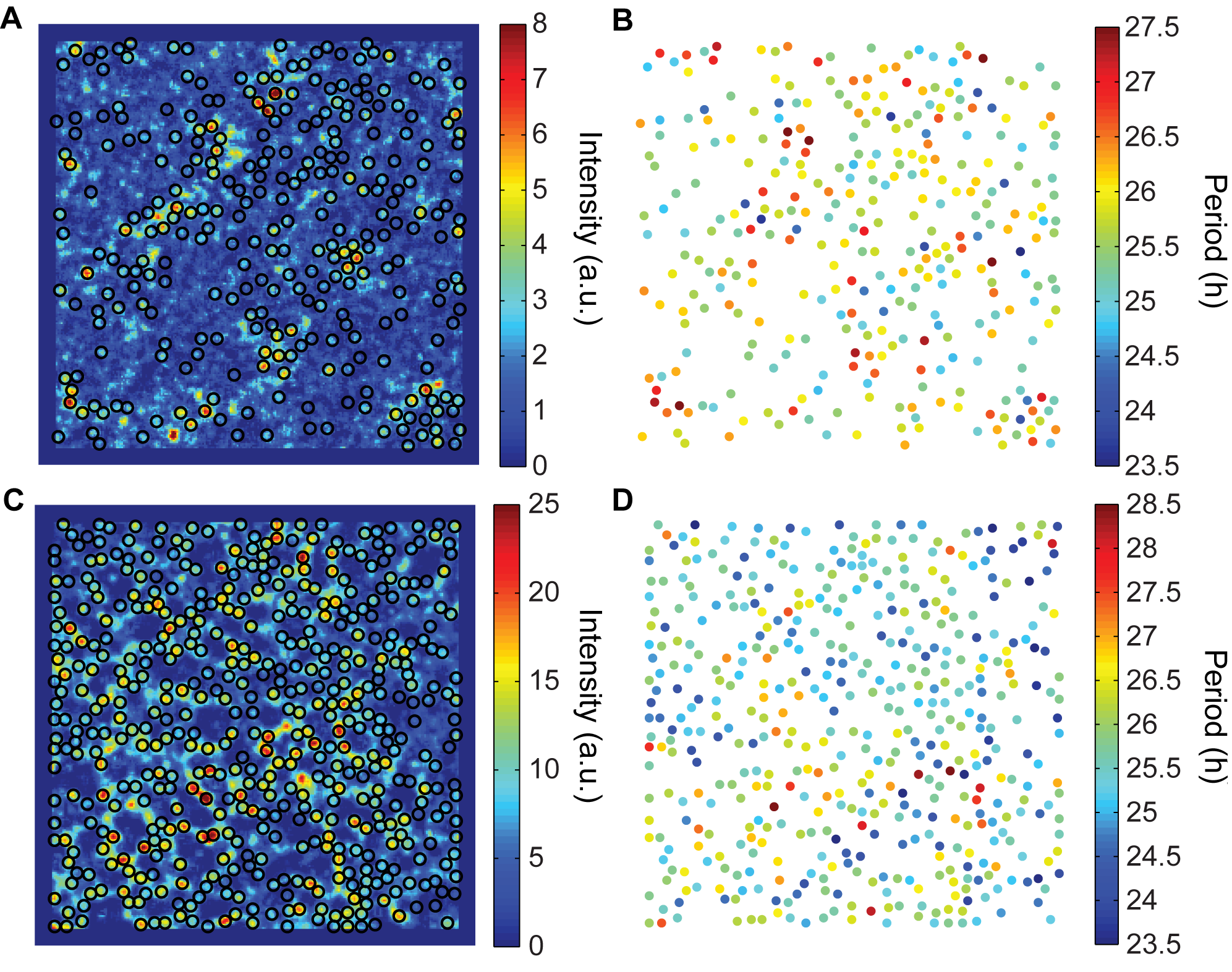

Supplement: Figure S1 — Examples of other cultures. A) Locations of 288 rhythmic ROIs in a WT Per2Luc hepatocyte culture with average local density around cells of roughly 12 cells/mm2. C) Locations of 497 rhythmic ROIs in another WT Per2Luc culture with average local density around cells of roughly 15 cells/mm2. B,D) Periods of the ROIs in (A) and (C). (TIF) [file pone.0087573.s001.tif]

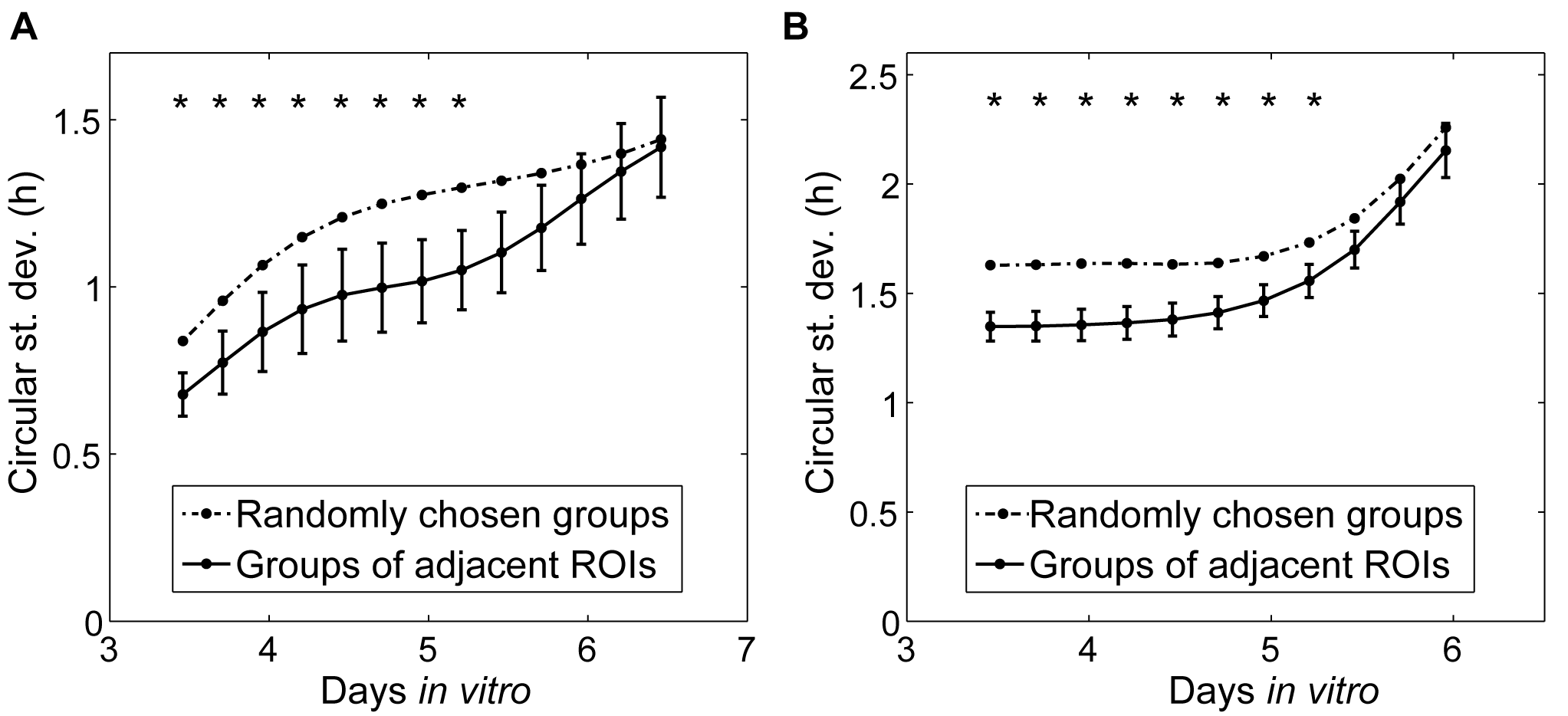

Supplement: Figure S2 — Additional examples of localized phase clustering. A) Circular standard deviation over time in groups of adjacent ROIs compared to groups of randomly selected ROIs for the culture shown in Figure S1A. The 12 groups of adjacent ROIs include all possible groups of 7 ROIs lying within a 325-µm diameter circle (ROIs are 78 µm or larger in diameter). B) Circular standard deviation over time in 54 groups of adjacent ROIs compared to randomly selected groups for the culture shown in Figure S1B. In both figures, asterisks mark time points at which the mean is significantly different (α = 0.05) according to a one-sample z-test for which 1,000,000 randomly selected groups were sampled to approximate the population distribution. (TIF) [file pone.0087573.s002.tif]

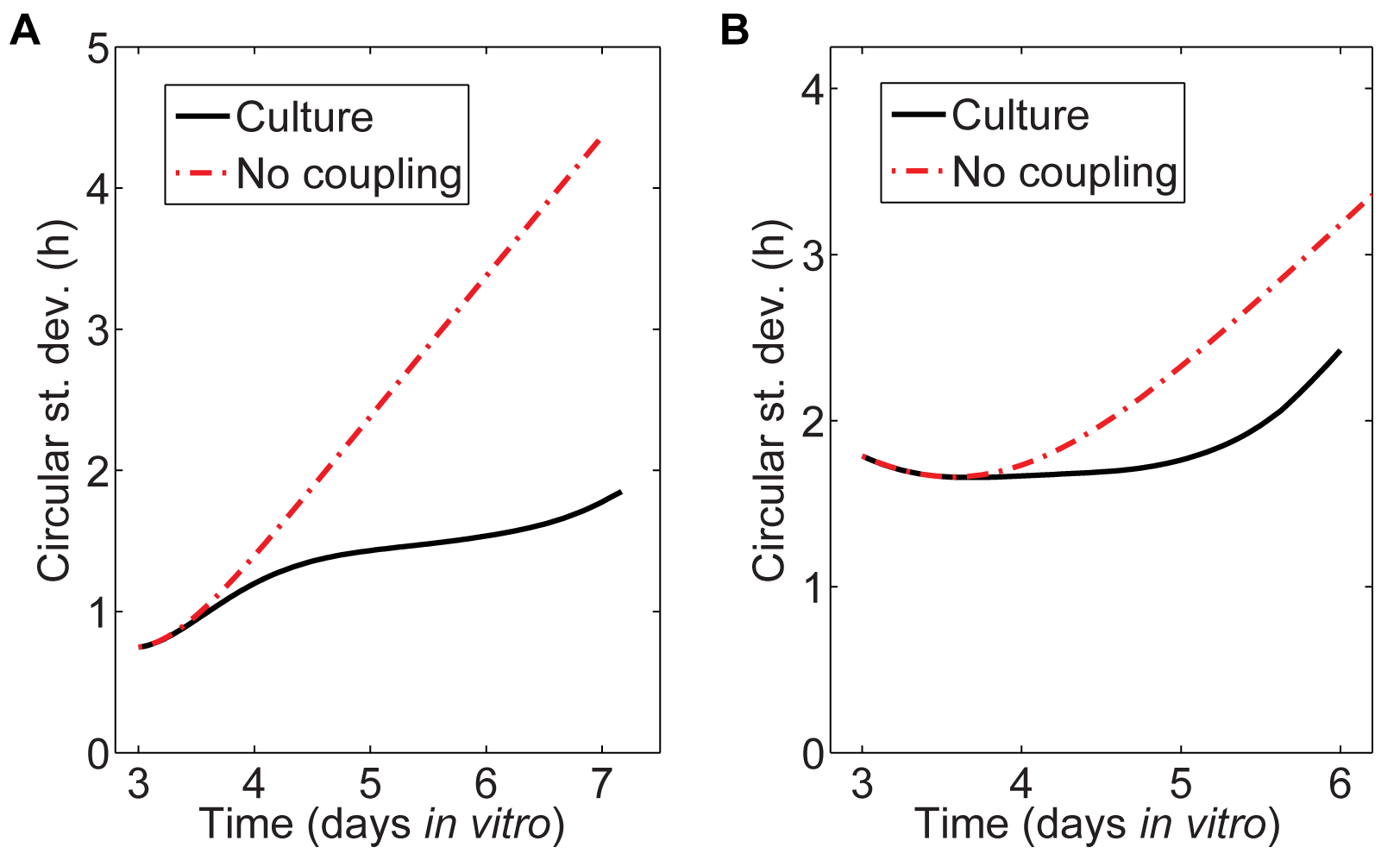

Supplement: Figure S3 — Additional examples comparing cultures and simulations. A) Comparison of circular standard deviation in observed ROI phases over time with that expected if no coupling were present (corresponding to C = 0 in the model) for the culture shown in Figure S1A. B) Similar comparison for the culture shown in Figure S1C. In both graphs, the initial phases and periods of the cells in the model are set equal to those on DIV 3 for the culture, so the phases in the model and culture agree initially but diverge after roughly 12 h. (TIF) [file pone.0087573.s003.tif]

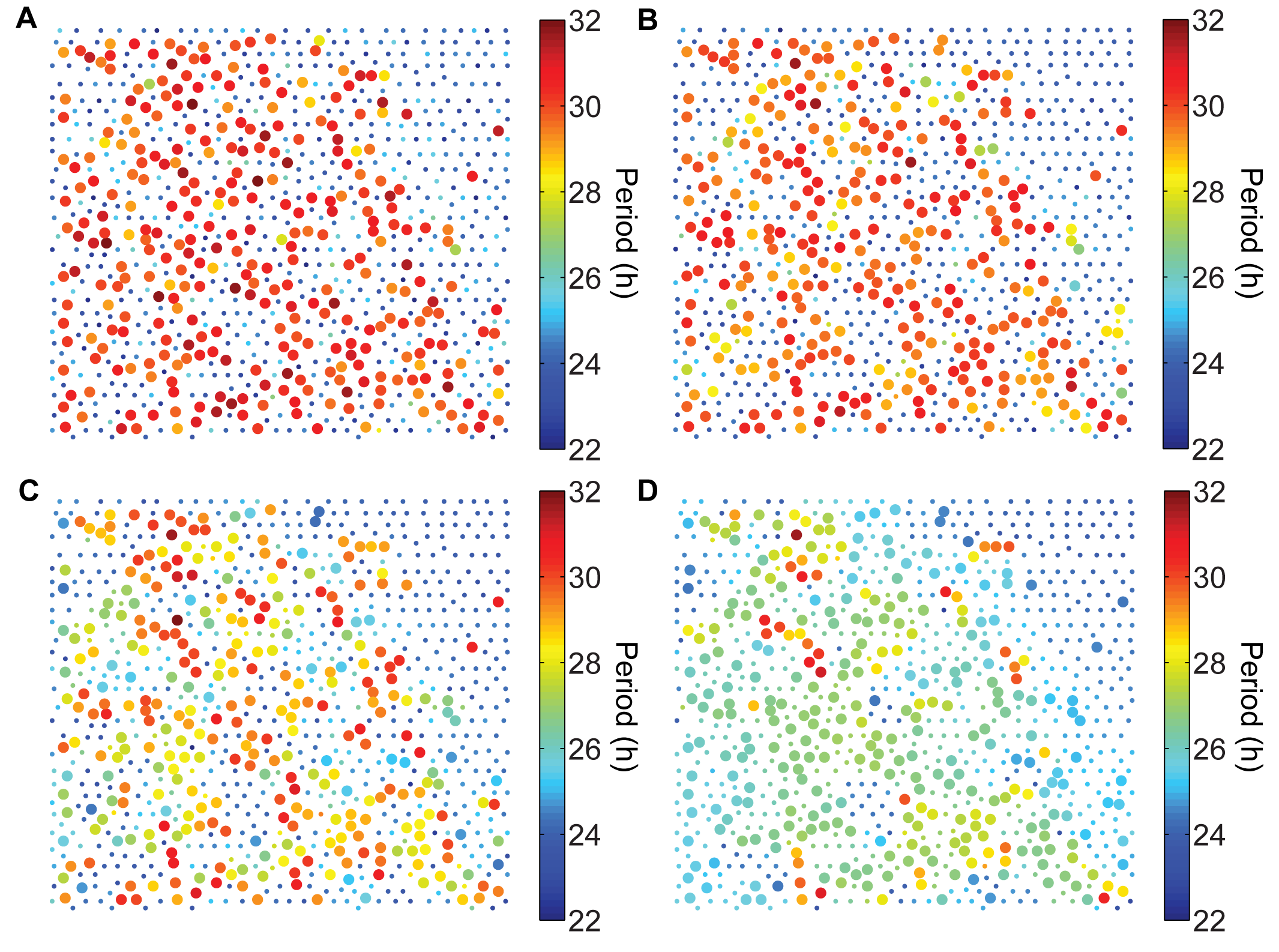

Supplement: Figure S4 — Periods of ROIs in mixed culture simulated with different coupling strengths. A) Periods of ROIs at end of 2-week simulation of Cry2−/− Per2Luc mixed culture with no coupling (C = 0). Resulting period of summed rhythm of Cry2−/− Per2Luc ROIs is 30.2 h. B) Simulation with C = 0.008, resulting in Cry2−/− Per2Luc summed rhythm period of 29.8 h. C) Simulation with C = 0.016, resulting in Cry2−/− Per2Luc summed rhythm period of 29.5 h. D) Simulation with C = 0.032, resulting in Cry2−/− Per2Luc summed rhythm period of 26.8 h. In all simulations, the 313 Cry2−/− Per2Luc ROIs (large circles) are located as shown in Figure 5, with periods and initial phases as measured from the culture. 583 ROIs mimicking WT (small circles), with period 24.0±1.0 h, were added to simulate a mixed culture. Local coupling has the same form as simulations in Figure 7. However, the local density of cells in these simulations is relatively high, as the spatial distribution of ROIs lacks the physical gaps inherent in the experimental cultures. (TIF) [file pone.0087573.s004.tif]
